# Practical Notes and Formulæ

**Published:** 1880-10-20

**Authors:** 


### PRACTICAL NOTES AND FORMULÆ.

**The Treatment of Neurasthenia, or Chronic Nervous Exhaustion.**—As to the medicinal agents which may be employed in these cases, I have found nothing that answers the purpose better than the milder bromides, associated with digitalis, in some such combination as the following:

℞. Sodii bromidi................................ ʒ vij

Acid. bromohyd. (Squibb's strong)................ ʒ iij

Tr. digitalis................................ ʒ iij

Bismuth. subnit................................ ʒ iv

Syr. prun. virg................................ ℥ vij—M.

S.—Shake well before using; one or two teaspoonsful in water, before or after meals.

In this prescription, as you will observe, the patient gets a small dose of the bromide of sodium, which is rendered more acceptable to the stomach, if not more useful, by the addition of bromohydric acid, especially in dyspeptic neurasthenias. The value of the prescription is enhanced by the addition of bismuth in some cases. If the acid is of the stronger kind, it should be given in proportionately smaller doses than the weaker. In such a mixture as I have offered, I have reason to think partial decomposition of the subnitrate of bismuth occurs, so as to afford, probably, an acid bromide of sodium and of bismuth combined.—DR. JEWELL, *in Chicago Med. Journal*

**Vaselin in Gynæcology.**—For making vaginal examinations, introduction of the speculum or manual exploration, Dr. De Snity recommends vaselin as the best suited for lubricating purposes. Oil and wax are likely to become rancid, and the putrefaction process cannot be arrested by the use of carbolic acid. Carbolized oil soon acquires an offensive odor, due to decomposition. Dr. De S. thinks that even soap is objectionable. Vaselin does not undergo any decomposition, and it is a soothing agent. Carbolized vaselin is better than the pure substance, and it may be used not only to anoint the finger, but as a topical application in case of certain ulcerations of the neck of the uterus. Vaselin may be mixed with potassium iodide, iodine or with belladonna. The following formulæ have been used by Dr. De S. with success:

℞. Carbolic acid................................ 1-00
Vaselin................................ 50-00

℞. Iodine................................ 1-00

Potassium iodide................................ 4-00

Vaselin................................ 30-00

—*Le Progres Medical.—Physician and Surgeon.*

**To Keep Flies from Horses.**—A cold infusion of walnut leaves sponged on the horse is said effectually to keep flies away.—*Louisville Med. News.*

Dr. I. T. Suggs, of Texas, writes: I will give a small sketch of a case, such as I have never seen described. June 16th, 1876, Mrs. M., a young maıried woman, one child twelve or thirteen months of age, had suffered a great deal in her confinement; says she had an abscess that discharged large quantities of pus for a length of time shortly after her confinement. From her history of the case, I suspected uterine disease. Upon making a metroscopic examination, I found no uterine disease, but at the point in the vagina, where I suppose vesico vaginal fistula occurs, was a remarkable ill-conditioned ulcer—no appearance of pus—had had no purulent discharge for several months—her general health was extremely bad. By cauterizing the ulce frequently with nitrate of silver, and strict constitutional treatment, she regained her health perfectly.

**Treatment of Poisoning by Strychnia.**—If the patient is seen in time for an emetic to be of service, give forty grains ipecac—not tartar-emetic, or any irritant emetic—and follow immediately with large draughts of plain, warm water; place the patient in a warm bath and encourage emesis by tickling the fauces. If the patient is already in convulsions, administer chloroform by inhalation until the spasms are subdued, then give the ipecac and warm water. As soon as the emetic has acted give the following:

| | | |
|---|---|---|
| ℞ | Hyd. chloral | ℈j-ij |
| | Bromide lithium | gr. x-℈j |
| | Simple syrup | ℥j M |
| Ft. sol. | | |

Sig.—Give at one dose, in a little cool, sweet water.—*Med. Rep.*

**Dr. Bartholow's Prescription for Asthma.**—

| | | |
|---|---|---|
| ℞ | Potass. iod | gr. xv |
| | Potass. bromid | gr. xx |

M. S. Take every four or six hours.—*Chicago Med. Review.*

**Surgical Treatment of Epistaxis.**—Thurston depends upon the well-known fact that liquid injected into one nostril returns by the other, and in cases of epistaxis introduces the nozzle of a syringe into the nostril not bleeding, and holds it firmly. A stream of cold water thrown in thus washes out all of the clots from the bleeding nostril, and often arrests the bleeding. If not efficient for this purpose, he uses a dilute solution of perchloride of iron.—*Brit. Med. Journal.*

**Chloroform Cough Mixture.**—This is prepared as follows:

| | | |
|---|---|---|
| ℞ | Morphia acet | gr. iij |
| | Tinct. belladonnæ | ʒij |
| | Spts. chloroformi | ʒvi |
| | Syr. senegæ | ℥j |
| | Syr. pruni virg. ad | ℥iv |

Dose, one teaspoonful three times per day.

**Nervines.**—An excellent remedy to procure rest in nervous and hysterical subjects is the following :

℞ Gum assafœtida........................................................ ʒj

Ext. hyoscyami.......................................................... gr. x

Ft. pills No. xx.

One to be taken at bed time.

or ;

℞ Tinct.....................................................................
Tinct. valerian...........................................................
Camph. water.......................................................aa ʒj

Dose, one tablespoonfull.

**Purgative Coffee.**—The following decoction is recommended in *Progres Medical* as an agreeable purge :

℞ Senna.................................................................. ʒ v

Ro1sted coffee........................................................ ʒ iiss

Boiling water.......................................................... ℥ iij

Milk ...................................................................... ℥ iv

Sugar ..................................................................... ℥ j

To be taken in a single dose by an adult.

We may remark that the same end is secured by simply mixing the proper dose of infusion or extract of senna with an ordinary cup of coffee. The taste of the drug is completely disguised, and its action is promoted by the coffee.

**Calcium Salicylate in the Serous Diarrhœa of Infants.**—Dr. Alexander Hutchins, in Med. Soc., county of Kings, recommends in strong terms calcium salicylate in serous diarrhœa. He suggests the following formula for administering the remedy :

℞ Acid salicylic.......................................................... gr. xxij
Creta preparata.......................................................... gr. viij
Mix and divide in chart No. vi.

One every two to four hours.

**Whooping Cough.**—Dr. Perry, in Brief, says: I have always found the following prescription to relieve my patients suffering with whooping cough :

℞ Ext. belladonna.......................................................... 1 grain.
Pulv. aluminis..........................................................20 grains.
Syr. zingib............................................................... 1 ounce.
Aq. cinnamon, q. s...................................................... 2 ounces.

M.—Sig. : Teaspoonful morning, noon, evening aad at bedtime, to a child ten years of age.

The effects of the belladonna must be watched if the susceptibility of the patient be greater than anticipated.

**A Pad for Pertussis.**—E. J. Beal, M. D., in the St. Louis Clinical Record, reports success in several cases with the following treatment:

Three or four thicknesses of white flannel, four by four inches, are quilted together and suspended by tape around the neck of the little sufferer, like a necklace. The pad so formed is to be saturated three or four times daily with the following mixture, and to be worn night and day. When the child sleeps, the pad to be placed in close proximity to the respiratory outlets:

| | | |
|---|---|---|
| ℞ | Ol. sassafras | ʒ iii. |
| | Ol. terebinth | ʒ iv. |
| | Fl. ext. belladon | ʒ ss. |
| | Phenol sodique | ℥ iv. |

It is apprehended that the little sufferer will place the pad in the mouth, it would likely be well to be guarded in not exceeding the amount of belladonna suggested.—*Medical Times.*

**Prescription for the Gouty Diathesis.**—We give below an elegant prescription suitable for constant use by persons suffering from troubles arising from the gouty diathesis. We are indebted to Prof. J. S. Welford, M. D., of this city, for the same, whose large experience in this class of affections makes his opinion valuable. This will be found very agreeable and efficacious, while it will be a substitute for many high-priced waters. This was intended at first as a substitute for the Weilbach water, which it seems to equal fully:

| | | |
|---|---|---|
| ℞ | Carbonate sodæ | gr. iiiss. |
| | Carbonate Lithia | gr. iss. |
| | Carbonate acid water | oj. |

M.—For thirst.—Sig., daily dose.—*Southern Clinic.*

**Aristocratic Remedy for Itch.**—

| | |
|---|---|
| Balsam of Peru | 1 ounce. |
| Benzoic acid | 110 grains. |
| Oil of cloves | 40 drops. |
| Alcohol | 2½ drachms. |
| Simple cerate | 7 ounces. |

Dissolve the essential oil and the benzoic acid in the alcohol, and mix them with the cerate. Lastly, add the balsam of Peru. It is said to effect a cure in twenty-four hours.—*Canada Medieal Record.*

**Ergot in Pharyngitis.**—The following has proved excellent in the treatment of chronic pharyngitis, where the blood vessels of the pharynx are enlarged and tortuous, and the secretions moderate:

| | | |
|---|---|---|
| ℞ | Ergotine | gr. xx. |
| | Tr. iodina, | f. ʒ i. |
| | Glycerine, | f. ℥ i. |

M.—S.—Apply to the pharynx freely twice daily, with a camel's hair brush.—*Physician and Patient.*

